# Long-Term Musical Training Alters Auditory Cortical Activity to the Frequency Change

**DOI:** 10.3389/fnhum.2020.00329

**Published:** 2020-08-21

**Authors:** Jihyun Lee, Ji-Hye Han, Hyo-Jeong Lee

**Affiliations:** ^1^Laboratory of Brain & Cognitive Sciences for Convergence Medicine, Hallym University College of Medicine, Anyang, South Korea; ^2^Department of Otorhinolaryngology, College of Medicine, Hallym University, Anyang, South Korea

**Keywords:** frequency change, spectral processing, musical training, N1/P2 auditory evoked potential, hemispheric asymmetry

## Abstract

**Objective**: The ability to detect frequency variation is a fundamental skill necessary for speech perception. It is known that musical expertise is associated with a range of auditory perceptual skills, including discriminating frequency change, which suggests the neural encoding of spectral features can be enhanced by musical training. In this study, we measured auditory cortical responses to frequency change in musicians to examine the relationships between N1/P2 responses and behavioral performance/musical training.

**Methods**: Behavioral and electrophysiological data were obtained from professional musicians and age-matched non-musician participants. Behavioral data included frequency discrimination detection thresholds for no threshold-equalizing noise (TEN), +5, 0, and −5 signal-to-noise ratio settings. Auditory-evoked responses were measured using a 64-channel electroencephalogram (EEG) system in response to frequency changes in ongoing pure tones consisting of 250 and 4,000 Hz, and the magnitudes of frequency change were 10%, 25% or 50% from the base frequencies. N1 and P2 amplitudes and latencies as well as dipole source activation in the left and right hemispheres were measured for each condition.

**Results**: Compared to the non-musician group, behavioral thresholds in the musician group were lower for frequency discrimination in quiet conditions only. The scalp-recorded N1 amplitudes were modulated as a function of frequency change. P2 amplitudes in the musician group were larger than in the non-musician group. Dipole source analysis showed that P2 dipole activity to frequency changes was lateralized to the right hemisphere, with greater activity in the musician group regardless of the hemisphere side. Additionally, N1 amplitudes to frequency changes were positively related to behavioral thresholds for frequency discrimination while enhanced P2 amplitudes were associated with a longer duration of musical training.

**Conclusions**: Our results demonstrate that auditory cortical potentials evoked by frequency change are related to behavioral thresholds for frequency discrimination in musicians. Larger P2 amplitudes in musicians compared to non-musicians reflects musical training-induced neural plasticity.

## Introduction

Understanding speech and other everyday sounds require the processing of the temporal and spectral information in sounds. Psychoacoustically, pitch perception is the ability to extract the frequency information of a complex stimulus. It relies on spectral cues because it requires the mapping of frequencies onto meaningful speech or music (Stangor and Walinga, 2014). In both speech and music, pitch provides spectral information to facilitate the perception of musical structure and the acquisition of speech understanding inferred from the pitch contour and prosody information (Moore, 2008; Oxenham, 2012). Pitch processing is even more crucial for understanding sounds under adverse listening conditions such as background noise (Fu et al., 1998; Won et al., 2011). Difficulties with listening in noise have been attributed to the reduced ability to segregate the spectral cues and noise (Gaudrain et al., 2007).

Attempts to demonstrate a relationship between frequency coding and music perception have been made to investigate the neural activities underlying the auditory function of people who have undergone musical training. There is a large body of literature on assessing whether long-term musical training affects perceptual changes in frequency coding (Shahin et al., 2003; Micheyl et al., 2006; Deguchi et al., 2012; Liang et al., 2016) since examining the neural processing of sounds in musicians can provide a conceptual model of auditory training. Several studies have reported a strong relationship between musical training and speech perception. For example, the acquisition of a foreign language can be facilitated by musical training due to the enhanced neural encoding for speech relevant cues such as formant frequencies of speech (Intartaglia et al., 2017). Musical training in early life produces an even greater influence on both neural and behavioral speech processing than in adult life. It has been revealed that young children engaged in piano training have enhanced cortical responses to pitch changes, and the neural changes are associated with their behavioral performances in word discrimination (Nan et al., 2018). These improvements suggest a link between musical training and functional and structural changes in the human brain. Neuroimaging studies have provided converging evidence that the volume of the brain regions related to speech processing is larger in musicians compared to non-musicians, which indicates neurophysiological changes occurred by training-induced brain plasticity (Schneider et al., 2002; Gaser and Schlaug, 2003; Bermudez et al., 2009; Hyde et al., 2009). These findings indicate that music and speech processing rely on partially overlapping neural and cognitive resources.

Perceiving music requires listeners to integrate various sources of sound information, including pitch, timbre, and rhythm, and these musical features are linked to cognitive/perceptual processing at the cortical level. Previous studies have found that musical training can change various auditory functions, including sound discrimination (Zuk et al., 2013), listening to a foreign language (Marques et al., 2007), as well as auditory attention (Seppänen et al., 2012). These studies have also demonstrated that the neural changes underlying the perceptual and cognitive changes as a function of musical training can be reflected in cortical responses to complex stimuli (Pantev et al., 1998; Shahin et al., 2003, 2007). In general, musicians show greater N1/P2 and late positive responses to musical and tone stimuli compared to non-musicians. The improved cortical activities in musicians exhibit experience-driven neural changes (Shahin et al., 2003; Marques et al., 2007; Seppänen et al., 2012). However, some studies have suggested that the neuroplasticity evidence in musicians may be an innate property in people whose auditory function is superior to others rather than music experience-driven factors (Schellenberg, 2015, 2019; Mankel and Bidelman, 2018).

Cortical N1/P2 responses can be elicited by changes in various types of sound: speech (Han et al., 2016), tonal (Martin and Boothroyd, 2000), and noise (Bidelman et al., 2018). Using frequency-modulated tonal stimuli, it has been found that the N1/P2 responses vary depending on the rate and the magnitude of frequency (Dimitrijevic et al., 2008; Pratt et al., 2009; Vonck et al., 2019). Furthermore, N1/P2 responses to frequency change are enhanced by long-term auditory training. For instance, a recent study by Liang et al. (2016) found that N1/P2 amplitudes are enhanced with an increase in the magnitude of frequency changes, and this was more evident in musicians compared to non-musicians. However, in their study, no relationship was found between behavioral performance for frequency change detection and the N1/P2 response measures.

Although there has been extensive research on the subject (Koelsch et al., 1999; Schneider et al., 2002, 2005; Bidelman et al., 2014; Hutka et al., 2015) which indicates that musical training can change neural processing to spectral change, the idea that altered neural responses are induced by long-term musical training or by other environmental/congenital properties is still controversial. Given that tracking the frequency pattern of tone and extracting the pitch of musical sounds both rely on spectral processing in the auditory cortex, an attempt to assess the neural sensitivity of musicians to subtle frequency changes can provide knowledge about the underlying auditory processing for music and speech. Therefore, we examined how the central auditory system of musicians encodes frequency information differently and related this to their behavioral perception ability. It is important to determine whether relationships exist between behavioral performance and objective cortical activities since it would indicate that cortical responses evoked by frequency change can be used as a marker for behavioral frequency discrimination. To answer the research question, we applied base frequencies of 250 and 4,000 Hz, because a previous study reported that cortical responses elicited by stimuli with the frequencies had a strong relationship with psychoacoustical thresholds of frequency discrimination (Dimitrijevic et al., 2008). We hypothesized that the cortical activity to frequency change is more enhanced in musicians compared to non-musicians. We further predicted that behavioral thresholds and the duration of musical training relate to the measures of cortical activity. Also, we measured the behavioral frequency discrimination both in quiet and noise conditions to associate with cortical responses, because musical training has improved sound in noise perception ability (Parbery-Clark et al., 2009b; Yoo and Bidelman, 2019). We assumed that musicians reveal better noise perception than non-musicians, indicating the musician’s advantage on the sound in noise perception.

## Materials and Methods

### The Participants

A total of 13 (six male) musicians [mean age ± standard deviation (SD) = 27.1 ± 5.0 years, all right-handed] and 11 (six male) age-matched non-musicians (mean age ± SD = 26.8 ± 5.31 years, all right-handed) participated in this study. We ran a two-sample *t*-test to examine whether our findings were driven by age. Results showed no significant difference in the age of musician and non-musician groups (*p* = 0.904), suggesting that age is not a contributing factor. All musicians reported that they had been receiving professional musical training for over 10 years regularly and received musical training at least three times a week during the training period. The types of musical training were vocal, piano, drum, haegeum, guitar, and violin. Details on musical training of the musicians are provided in Table 1. All participants were recruited through online advertising and were compensated for their participation. Both groups had normal pure-tone thresholds below 20 dB hearing loss (HL) at octave test frequencies from 250 to 8,000 Hz, and they had no history of neurological or hearing disorders. The study protocol was approved by the Institutional Review Board of Hallym University Sacred Hospital, Gangwon-Do, South Korea (File No. 2018-02-019-001), and written informed consent were obtained from each participant.

**Table 1:** Characteristics of musicians.

Subjects	First instrument	Age began musical training	Secondary instrument	Age began musical training	Years of musical training
**Mus 1**	Vocal (Korean tradition)	14			20
**Mus 2**	Piano	9			12
**Mus 3**	Piano	9			11
**Mus 4**	Piano	6	Vocal	17	20
**Mus 5**	Bass guitar	20			16
**Mus 6**	Piano	6	Cello	25	19
**Mus 7**	Piano	5	Vocal	23	25
**Mus 8**	Haegeum (Korean tradition)	16	Vocal	24	10
**Mus 9**	Piano	8	Clarinet	16	20
**Mus 10**	Drum	17		17	12
**Mus 11**	Guitar	10			10
**Mus 12**	Violin	10	Piano	10	13
**Mus 13**	Guitar	13			13

### Psychoacoustics: Frequency Discrimination Test

Frequency discrimination was applied as a standard adaptive, three-interval, three-alternative, forced-choice, two-down, one-up procedure to detect the threshold for each subject. The base frequencies used for the frequency discrimination test were 250 and 4,000 Hz. During each trial, two of three intervals contained base frequency pure tones, while the remaining one had a pure tone with a higher frequency change. Individual tones were 300 ms in duration and separated by an inter-stimulus interval of 500 ms. The three intervals were presented randomly. The initial differences in frequency between the base and the change were 50 and 100 Hz for 250 and 4,000 Hz tones, respectively. The step size from the second trial was 12 Hz for 250 Hz and 50 Hz for 4,000 Hz. The difference was decreased or increased for the subsequent trial depending on whether there were two consecutive correct responses or a single incorrect response, respectively. When the current step size was larger than the difference, it was varied to half the difference. Each condition was ended after a maximum of 60 trials or 12 reversals. The averaged thresholds were measured with the last eight reversals to compute each subject’s difference limen (DL). Subjects were instructed to find the frequency change among the three interval choices by clicking a mouse on a computer screen. For a noise condition, four types of background threshold-equalizing noise (TEN) were used; no TEN and +5, 0, and −5 dB signal-to-noise ratio (SNR) TEN. These noise conditions for each base tone were randomly presented to avoid the carryover effect (Logue et al., 2009; Dochtermann, 2010; Bell, 2013). During the testing, the subjects were seated in a sound-attenuated booth, and sound stimuli were presented through 2 channel speakers at a level of 70 dB HL.

Outliers were determined based on the interquartile range (IQR) method (Kokoska and Zwillinger, 2000). The outliers were identified by defining limits on the sample values that are a factor *k* of the IQR below the 25th percentile or above the 75th percentile. We used 3 for *k* to identify values that are extreme outliers. None of the musicians were defined as an outlier while two non-musicians were rejected.

### Electroencephalogram (EEG) Acquisition and Analysis

#### Stimuli and Experimental Procedure

**Figure 1:**
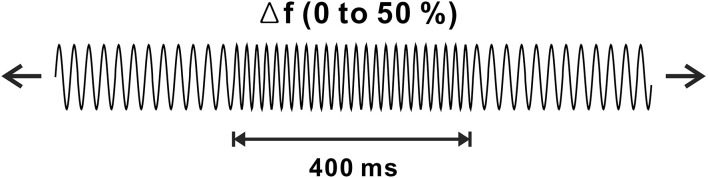
Schematic representation of frequency change stimulus. Continuous tones with base frequencies of 250 or 4,000 Hz are presented with occasional changes in frequency change of 10%, 25%, or 50% lasting 400 ms.

Stimuli for the frequency change and experimental procedures were based on a previous frequency change experiment (Dimitrijevic et al., 2008). Auditory stimuli were generated in MATLAB (MathWorks, Inc., Natick, MA, USA), and they were sampled at a rate of 48,828 Hz. Frequency change stimuli were constructed using two continuous base tones, 250 and 4,000 Hz, each with upward frequency changes of 10%, 25%, or 50% for 400 ms. The order of the frequency changes was randomly determined. The intensity of the frequency change was equated to equal loudness concerning the base frequency. The ongoing stimuli consisted of frequency change stimuli followed by base frequency tones varied from 1.6 to 2.2 s to prevent anticipating the point where the frequency change occurred. To avoid a transient click, which was produced when changing the stimuli, we manipulated the stimuli to occur at the zero phase. Figure 1 shows a schematic of the frequency changes of the stimulus. A minimum of 100 trials for each frequency change was presented in two blocks. The total electroencephalogram (EEG) recording time for each subject was approximately 30 min, during which the subjects were seated in a comfortable reclining chair and watched a close-captioned movie of their choice while the frequency change stimuli were presented through 2 channel speakers located 1.0 m away from the subject.

#### EEG Acquisition and Data Processing

Multi-channel EEG data were acquired using the actiCHamp Brain Products recording system (Brain Products GmbH, Germany). Scalp potentials were recorded at 64 equidistant electrode sites, all electrodes were referenced to the reference electrode, electrical impedances were reduced below 10 kΩ, and EEG signals were amplified and digitized at 1,000 Hz. During the EEG recording, continuous data were band-pass-filtered from 0.1 to 120 Hz and a notch filter for 60 Hz noise was applied.

#### EEG Data Analysis

All EEG data were preprocessed offline using Brain Vision Analyzer 2.2 (Brain Products GmbH, Germany). Continuous eye blink and horizontal movement artifacts were rejected using the independent component analysis (ICA) algorithm. After the ICA correction, the data were further analyzed in MATLAB. Continuous EEG data were down-sampled to 250 Hz and band-pass-filtered from 0.1 to 40 Hz. The data were segmented from −100 to 400 ms with 0 ms at the onset of frequency change. Segmented data were baseline-corrected from −100 to 0 ms and re-referenced to an average reference. Separate averages for individual frequency changes were also performed. Peak detection was performed for N1/P2 on the frontal central electrodes located at the near vertex. N1 peaks were determined as the first negative potentials between 70 and 150 ms after stimulus onset, while the most positive potentials between 120 and 230 ms were defined as P2 peaks.

#### Dipole Source Analysis

This was performed using BESA Research 7.0 (Brain Electrical Source Analysis, GmbH, Germany), as described previously (Han et al., 2016). The source analysis was performed on individual averaged waveforms with band-pass filtering (0.5–40 Hz, 12 dB/octave, zero-phase). In the first step, two symmetric regional dipole sources were inserted near the auditory cortical regions. For N1 and P2 dipole fitting, the mean area over a 20 ms window around the N1 and P2 peaks on the global field power was used for further analysis. The dipole source activities were allowed to vary in location, orientation, and strength, and the maximum tangential sources were fitted on the N1 and P2 peaks. The residual variance was examined for each 20 ms window, for which all subjects obtained 5% or less variance. Statistical differences in the grand mean source waveforms were assessed across the different conditions and subject groups.

### Statistical Analysis

For the behavioral thresholds, the main effect of the subject groups (musician vs. non-musician), the noise (+5 SNR, 0 SNR, and −5 SNR; for noise condition only) and base frequency (250 and 4,000 Hz) settings were examined using repeated-measures analysis of variance (rmANOVA) for quiet and noise condition, separately. rmANOVA was used to assess the main effects of frequency change (10%, 25%, and 50%), the base frequency for within-subject comparison on the cortical measures (the frequency change and the base frequency were set as continuous variables). For between-subject factors, musician and non-musician groups were included. We performed this analysis using the fitrm and ranova functions in MATLAB. *Post hoc* testing was applied using Tukey’s honestly significant difference tests, and paired *t*-tests were conducted for group comparisons. Pearson’s product-moment correlation coefficient was applied to assess relationships among the behavioral measures and demographic factors with the electrophysiological measures. Multiple pairwise comparisons were adjusted with the false discovery rate (FDR). All data are expressed as the mean ± standard error (SE) unless otherwise stated.

## Results

### Behavioral Frequency Discrimination

**Figure 2:**
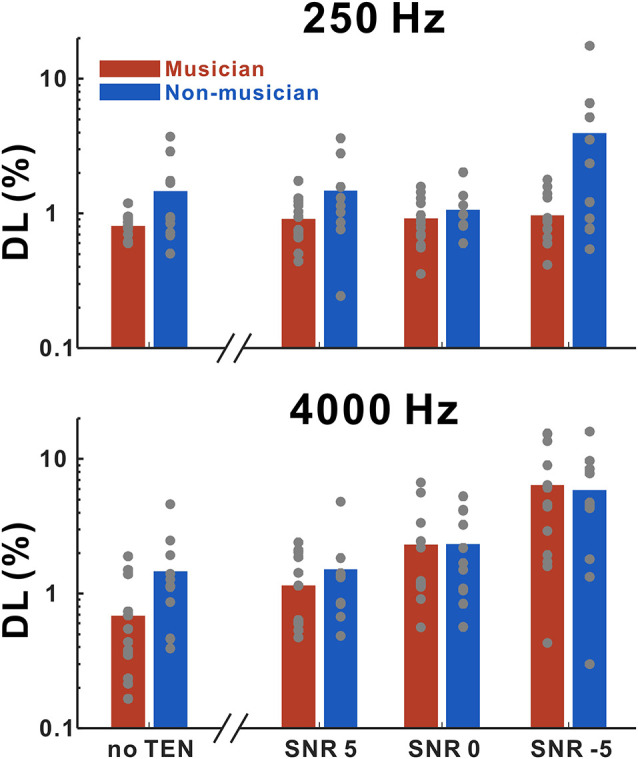
Mean frequency discrimination thresholds for 250 and 4,000 Hz in musicians and non-musicians as a function of listening conditions including no threshold-equalizing noise (TEN), SNR +5 dB, SNR 0 dB, and SNR −5 dB. Note that gray dots indicate each subject. Musicians show decreased thresholds compared to non-musicians for both 250 and 4,000 Hz in no TEN condition.

Figure 2 shows frequency discrimination thresholds for 250 and 4,000 Hz as a function of listening conditions. We performed rmANOVA to examine the main effects of group, base frequency, and noise level (for the noise condition only) for quiet and noise conditions, separately. In the quiet condition, the results revealed significant main effects of the groups (*F*_(1,20)_ = 5.18; *p* = 0.034) in that the thresholds in the musicians were lower than those in the non-musicians. In the noise condition, a significant interaction between noise level and base frequency (*F*_(2,34)_ = 64.75; *p* < 0.0001) was found. Tukey’s HSD (honestly significant difference) test showed that the thresholds at 250 Hz base frequency were significantly lower than those at the 4,000 Hz base frequency for −5 SNR (*p* < 0.0001) and 0 SNR (*p* = 0.0086) conditions. In addition, significant differences between +5 SNR and 0 SNR (*p* = 0.0168), +5 SNR and −5 SNR (*p* < 0.0001), and 0 SNR and −5 SNR (*p* < 0.0001) were found for the 4,000 Hz base frequency.

### Electrophysiology

#### N1/P2 Cortical Responses

**Figure 3:**
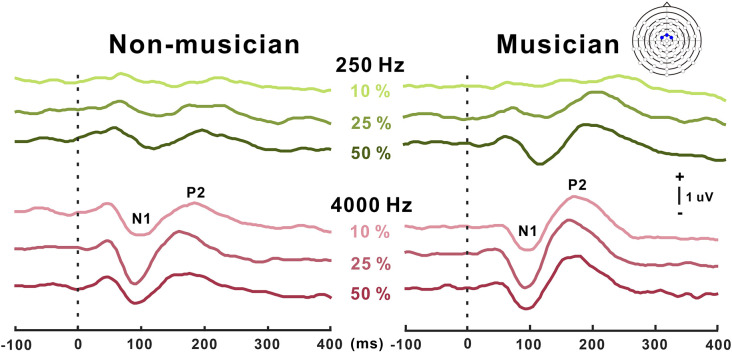
Grand average N1/P2 responses to frequency changes. N1/P2 cortical potentials to frequency change stimuli in non-musicians (left) and musicians (right). Green and red color waveforms represent cortical responses for 250 and 4,000 Hz base frequencies, respectively. The amount of frequency change is indicated as a percentage. In the top right, a figure shows an equidistant cap layout indicating the frontal central electrodes (blue dots).

Grand mean waveforms as a function of frequency change for non-musicians and musicians are given in Figure 3. In general, the N1/P2 cortical responses were modulated by frequency changes, and the modulations were more evident at 4,000 Hz and the musician group, compared with 250 Hz and the non-musician group.

**Figure 4:**
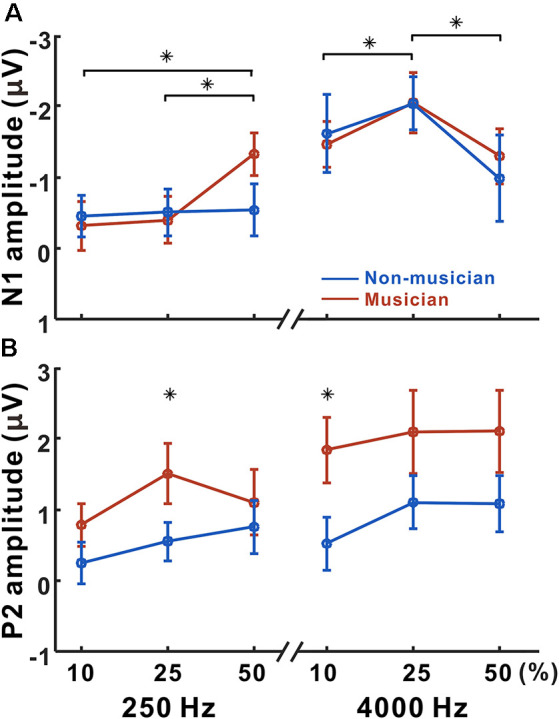
Mean N1 and P2 amplitudes as a function of frequency change. Mean of N1 **(A)** and P2 **(B)** amplitudes in musicians and non-musicians are shown. Note that significant differences among listening conditions are revealed for the N1 (250 Hz: 10 vs. 50, 25 vs. 50%, 4,000 Hz: 10 vs. 25, 25 vs. 50%), whereas the differences between musicians and non-musicians are found for the P2 amplitude (250 Hz: 25%, 4,000 Hz: 10%). Asterisks (*) indicate significant differences (*p* < 0.05).

Figure 4 shows N1 and P2 amplitudes as a function of frequency change starting at 250 and 4,000 Hz for the musician and non-musician groups. rmANOVA to examine the effect of frequency change on N1 response revealed a significant frequency change × base frequency interaction for N1 amplitude (*F*_(1,22)_ = 24.32; *p* < 0.0001) and latency (*F*_(1,22)_ = 54.43; *p* < 0.0001). The *post hoc* analysis confirmed that the N1 amplitude to the 50% change was larger than those to the 10% (*p* = 0.005), and 25% (*p* = 0.024) changes at the 250 Hz base frequency. The *post hoc* analysis also showed that the N1 amplitude to the 25% change was larger than those to the 10% (*p* = 0.038) and 50% (*p* < 0.0001) changes at the 4,000 Hz base frequency. In addition, the N1 amplitude for 4,000 Hz was significantly larger compared to 250 Hz with 10% (*p* < 0.0001) and 25% (*p* < 0.0001) frequency changes. For the N1 latency, the response at 4,000 Hz was significantly shorter than that at 250 Hz for all frequency changes (*p* < 0.0001). No significant group differences were found for N1.

Significant base frequency × frequency change interactions were found for P2 amplitude (*F*_(1,22)_ = 10.97; *p* = 0.003) and latency (*F*_(1,22)_ = 14.64; *p* < 0.0001). The *post hoc* results show that P2 amplitude to 25% change was greater than that to the 10% change for 250 Hz only (*p* = 0.004). For the P2 latency, the 25% frequency change elicited significantly shorter responses compared to the 10% change for 4,000 Hz (*p* = 0.019). Compared to 4,000 Hz, the P2 responses for 250 Hz significantly decreased in amplitude for 10% (*p* = 0.012), 25% (*p* = 0.022), and 50% (*p* = 0.015) frequency changes, while the latency increased for 10% (*p* < 0.0001), 25% (*p* < 0.0001), and 50% (*p* = 0.015). Significant differences between the musician and non-musician groups were found in the P2 amplitudes: that of the P2 in musician group was significantly larger than that of the non-musician (*F*_(1,22)_ = 6.58; *p* = 0.018). In addition, an interaction between the groups and frequency change was revealed (*F*_(1,22)_ = 4.67; *p* = 0.042). The *post hoc* results show that the P2 amplitudes of the musicians were greater than those of the non-musicians for 10% (*p* = 0.005) and 25% (*p* = 0.022) frequency changes. In addition, the P2 amplitudes for the 25% frequency change were greater than those for the 10% frequency change in both the musician group (*p* = 0.009) and the non-musician group (*p* = 0.029). No group differences were found for P2 latency.

#### Dipole Source Activity

**Figure 5:**
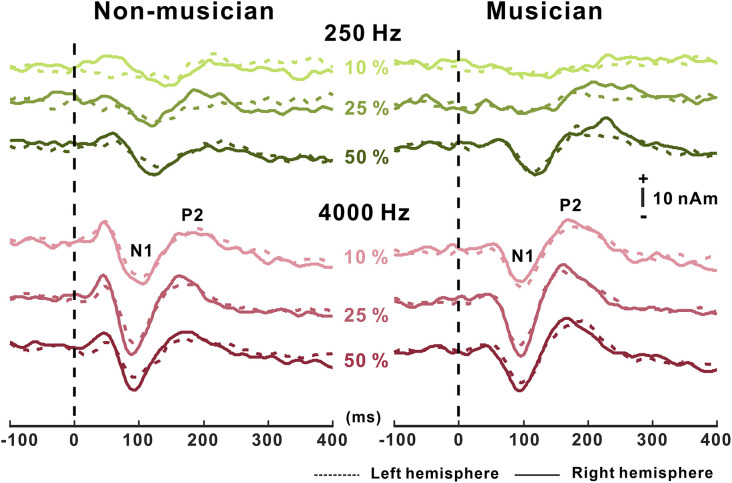
Dipole source waveforms to frequency changes in non-musician and musician groups. N1 dipole source waveforms to frequency change stimuli in non-musicians (left) and musicians (right). Green and red color waveforms represent dipole activity for 250 and 4,000 Hz base frequencies, respectively. Dashed lines of waveform represent dipole activity in the left hemisphere and solid lines indicate activity in the right hemisphere.

The grand average N1 dipole source waveforms as a function of frequency changes for the 250 and 4,000 Hz base frequencies are shown in Figure 5. Using two symmetric single equivalent dipoles, the N1/P2 dipoles were fitted, and amplitudes and latencies of N1/P2 sources waveforms were averaged for each hemisphere. The overall morphology of the N1 dipole waveforms was similar to the N1 scalp-recorded waveforms in that N1 activity increased as the frequency change became greater, which was more apparent at 4,000 Hz than 250 Hz. P2 dipole source analysis showed that the musician group had greater P2 dipole activity than the non-musician group.

A significant frequency change × base frequency × hemisphere interaction was found for N1 dipole amplitude (*F*_(1,22)_ = 6.45; *p* = 0.019). The *post hoc* results show that the N1 dipole amplitudes in the right hemisphere were greater than those in the left hemisphere for 25% (*p* = 0.019) and 50% (*p* = 0.031) changes at 4,000 Hz. Similar to the dipole amplitude, a significant frequency change × base frequency × hemisphere interaction for N1 dipole latency was revealed (*F*_(1,22)_ = 6.63; *p* = 0.017). The results show that the dipole latencies in the right hemisphere were shorter than those in the left hemisphere for 4,000 Hz with the 50% frequency change (*p* = 0.03).

For P2 dipole amplitude, two interactions including frequency change × hemisphere (*F*_(1,22)_ = 9.43; *p* = 0.006) and frequency change × base frequency (*F*_(1,22)_ = 114.04; *p* < 0.0001) were found. The P2 dipole amplitudes were greater in the right hemisphere than in the left hemisphere for 25% (*p* = 0.053) and 50% change (*p* = 0.019). Also, the P2 dipole amplitudes to 4,000 Hz were greater compared to 250 Hz for 10% (*p* = 0.005), 25% (*p* = 0.007), and 50% frequency changes (*p* = 0.029). For P2 dipole latency, a significant base frequency × frequency change interaction was found (*F*_(1,22)_ = 3,159; *p* < 0.0001) such that the P2 latencies for a 25% frequency change were significantly shorter than those for 10% at 4,000 Hz (*p* = 0.006). The P2 dipole latencies for 250 Hz were prolonged compared to 4,000 Hz for 25% (*p* = 0.002) and 50% (*p* = 0.004) frequency changes.

The effect of musical training on hemispheric asymmetry for spectral processing was examined by comparing left- and right-hemispheric activation separately between the musician and non-musician groups. For the group comparison, we conducted a two-sample *t*-test and found significant group differences for both left (*p* = 0.001) and right hemispheres (*p* = 0.013). The results indicate that P2 dipole source activities in both hemispheres of the musicians were larger than in non-musicians.

### Relationship Between N1/P2 Cortical Response and Behavioral Performance/Duration of Musical Training

**Figure 6:**
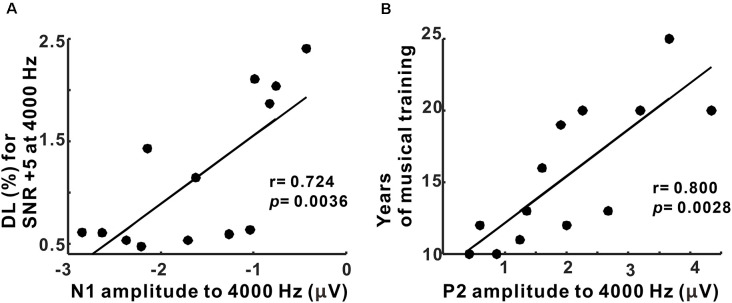
Correlations between behavioral performance/duration of musical training and N1/P2 amplitudes in musicians. **(A)** N1 amplitudes to 4,000 Hz condition are significantly related to frequency discrimination thresholds for SNR +5 at 4,000 Hz. **(B)** A significant relationship between P2 amplitudes to 4,000 Hz and the duration of musical training is revealed.

Pearson’s correlation results showed that the N1 amplitudes to 4,000 Hz were positively correlated with behavioral thresholds for 4,000 Hz with +5 SNR TEN (*p* = 0.0036, corrected for multiple comparisons; Figure 6A). Moreover, the P2 amplitudes to 4,000 Hz base tone were associated with the duration of musical training (*p* = 0.0028, corrected for multiple comparisons; Figure 6B). However, none of the latency measures were correlated with behavioral performance and musical training.

## Discussion

Our aim in this study was to examine the effect of musical training on behavioral frequency discrimination as well as N1/P2 cortical responses. These are elicited by tones with frequency change, and their relationships with the threshold for frequency discrimination and duration of musical training were assessed. Our results demonstrate that P2 was increased in musicians compared to non-musicians whereas N1 revealed more stimulus-dependent characteristics in that it was modulated by frequency change. The results of the dipole source analysis show that the N1/P2 dipole activity in response to the frequency change stimuli was greater in the right hemisphere, and the P2 dipole source activities in musicians were larger than those in the non-musicians for both hemispheres. Finally, the N1 and P2 amplitudes were related to behavioral performances and the duration of musical training, respectively.

### Effect of Musical Training on Behavioral Frequency Discrimination

In the behavioral frequency discrimination test, the thresholds of the musicians were lower than those of the non-musicians in the no TEN condition. These results indicate that the musicians were able to discriminate smaller spectral differences that the non-musicians could not, especially under quiet listening conditions. Previous studies assessing pitch discrimination in quiet conditions have reported relatively consistent results that musicians outperformed non-musicians in discriminating spectral features of stimuli, thereby confirming the better pitch perception of the former (Tervaniemi et al., 2005; Micheyl et al., 2006; Liang et al., 2016). Indeed, musical training leads to an enhancement in the ability to track frequency change and detect spectral cues in sounds. On the other hand, in the noise condition, the threshold for frequency discrimination in the musicians was not different from that in the non-musicians, which is similar to recent studies reporting that any advantage incurred by musical training on sound perception is questionable in the presence of noise-masking (Ruggles et al., 2014; Boebinger et al., 2015; Madsen et al., 2019). Meanwhile, it is still controversial whether musician advantage for auditory perception in noise exists or not. Studies in which behavioral tests were conducted on musicians have shown that musical training can improve speech-in-noise perception (Parbery-Clark et al., 2009a, 2012; Yoo and Bidelman, 2019). Furthermore, those studies have provided neurological evidence of better speech-in-noise perception by musicians (Musacchia et al., 2007; Parbery-Clark et al., 2011, 2012; Zendel et al., 2015; and reviewed in Coffey et al., 2017). However, in the current study, musical expertise for noise perception was not evident. One possible reason for this is related to the test paradigm and stimulus type used to evoke a response. In a study using speech with multiple maskers varied in content and similarity to speech, improved performances by musicians in a frequency discrimination task have been revealed, although this does not carry over to speech-in-noise perception (Boebinger et al., 2015). Similarly, Micheyl et al. (2006) and Ruggles et al. (2014) reported that musicians have an advantage in pitch discrimination that is not present for perceiving masked sounds. Another explanation for no effect of music training on sound in noise processing is that the musician benefits on the noise perception can be restricted to the specific sounds which are more linguistically and cognitively demanding. Several studies have suggested that the musician’s advantage in noise perception is dependent on the complexity of target sounds or tasks (Krizman et al., 2017; Yoo and Bidelman, 2019). For example, Yoo and Bidelman reported that musicians revealed improved sentences in noise perception, but the musician advantage was not applied for words in noise processing. In summary, the results of these studies suggest that the possible advantage of sound perception incurred by musical training is questionable in the presence of noise-masking, and it would be dependent on the complexity of the task (Ruggles et al., 2014; Boebinger et al., 2015; Madsen et al., 2019).

Investigating the neural overlap between pitch perception and perceiving sound in noise could uncover a mechanism to explain the perceptual advantages observed in musicians. Musical practice is a complex form of training consisting of dozens of perceptual and cognitive skills drawing on hearing, selective attention, and auditory memory. However, previous works examining the relationship between musical experience and cognitive/perceptual skills have shown that musical training is only related to specific musical features such as pitch, melody, and rhythm perception (Ruggles et al., 2014). Thus, selective listening related to the perception of masked sounds may not be a crucial aspect linked to musical training. Moreover, it has been suggested that the outcomes of musical training may not always be generalizable beyond the tasks that are closely related to musical perception (Geiser et al., 2009; Okada and Slevc, 2018). Rather, high sensitivity to sound in noise seems to be associated with daily music-related behavior, such as listening to music in everyday life (Kliuchko et al., 2015). Alternatively, the neurological basis for music perception is another possibility for the result as the cortical areas governing music and speech may not completely share their neural origins (Albouy et al., 2020; reviewed in Peretz et al., 2015).

### Effects of Musical Training on N1/P2 Cortical Potentials

#### N1 Modulation as a Function of Frequency Change

We found that N1 is modulated as a function of frequency change to a greater degree than P2, whereas P2 reflects the musical training-induced enhancements in the musicians. Previously, it has been suggested that the N1/P2 responses are evoked by acoustic changes in a sound: either amplitude (Han and Dimitrijevic, 2015), intensity (Dimitrijevic et al., 2009) or frequency (Shahin et al., 2003; Dimitrijevic et al., 2008; Pratt et al., 2009). The N1 was evoked by stimuli with changes in frequency which is close to the level of behavioral thresholds in frequency discrimination. In turn, the N1 may not be elicited by the sound with frequency change which is not detected by listeners perceptually (Martin and Boothroyd, 2000; Jones and Perez, 2001). In general, the N1 amplitude is modulated by an increase in frequency that is more apparent for stimuli with frequencies higher than 1,000 Hz (Picton, 2011). Enhanced amplitude with frequency increase has been attributed to the level of neuronal activation relating to the range in basilar membrane deflection (Rose et al., 1967; Picton, 2011).

In our study, N1/P2 amplitudes were larger for the higher frequency relative to the lower one. Although these were larger for the lower frequency compared to the higher one, some studies using the mismatch negativity (MMN) paradigm have reported similar results to our findings. For example, using a frequency change as a deviant stimulus, Novitski et al. (2004) found larger MMN responses to a higher frequency than a lower one. A possible reason for the increased cortical responses to the higher frequency could be related to the frequency change experiment including change stimuli embedded in the ongoing tones. This is similar to the MMN paradigm (Lavikainen et al., 1995) in which infrequent deviant stimuli presented with repetitive standard sounds. From this point of view, we speculate that the listening condition of the frequency change activates the neuronal populations in a similar way to the MMN paradigm (see the review in Alho, 1995).

#### P2 Response Reflecting Musical-Training Induced Plasticity

In contrast to N1, our results show that P2 responses to frequency changes in musicians are more robust than in non-musicians. Human electrophysiological studies have also shown enhanced P2 cortical activity in individuals with short-term auditory training (reviewed in Tremblay, 2007; Tremblay et al., 2014), language experience (Wagner et al., 2013), and short/long-term musical training (Atienza et al., 2002; Shahin et al., 2003; Tremblay, 2007; Tong et al., 2009). More recently, results showing increased P2 to trained pitch sounds during passive listening infer that training-induced cortical plasticity is related to permanent perception changes rather than the effect of selective attention (Wisniewski et al., 2020). There is growing neuroimaging evidence to support the notion that the neural representations of complex sounds such as music at the peripheral and central levels are influenced extensively by experience or training (Parbery-Clark et al., 2012; Sankaran et al., 2020). Perceiving music requires listeners to integrate sources of information, including amplitude, timbre, and pitch, each of which can provide cues for perceiving music. Neuroanatomically, the understanding of music requires systematic processing as a set of hierarchical neural representations in different areas of the brain. Previous studies have proposed that music perception requires the activation of multiple areas of the brain involved with not only sound discrimination but also cognitive/perceptual skills (Okada and Slevc, 2018). Among the long-latency responses, P2 is related to neural processes mediating cognitive/perceptual aspects of sound processing (Näätänen et al., 1993; Alain et al., 2007). Therefore, we assume that P2 represents training-induced cortical plasticity due to the characteristic of being sensitive to acoustic features importantly contributing to music perception.

In Neuromusicology, there has been a long-standing debate about whether the central auditory processing to musical features is altered by musical training (nurture) or preexisting factors (nature). Previous findings have supported the idea of experience-driven plasticity of musicians by showing that training changes the neural representation of acoustic features in individuals with extensive musical experience (Pantev et al., 2001; Shahin et al., 2008). It has been suggested that skilled musicians exhibit enhanced cortical representations of musical timbres associated with the instrument they have trained with (Pantev et al., 2001; Pantev and Herholz, 2011). Such timbre specificity constrained to the principal instrument supports the theory that changes in neural activity in musicians are mainly driven by experience (reviewed in Pantev and Herholz, 2011). Furthermore, in studies investigating the effect of short-term musical training on cortical plasticity, Tremblay et al. (2001) and Atienza et al. (2002) found that P2 responses were enhanced by short-term intensive training in non-musicians. These results indicate that auditory cortical responses can be altered regardless of the musical training duration of the non-musicians. Given that professional musicians receive much longer training than non-musicians, it can be inferred that the cortical plasticity induced by the training should be greater in musicians. Numerous studies have reported the musical training effects both the behavioral level (Shahin et al., 2003, 2008; Tervaniemi et al., 2005; Liang et al., 2016; Intartaglia et al., 2017) and the perceptual levels (Gaser and Schlaug, 2003; Bermudez et al., 2009; Hyde et al., 2009) of the auditory processing. Meanwhile, an attempt to explain the musician’s advantage of innate properties has been made. These studies have supported the view that genetic factors could be involved in the etiology of musical properties including absolute pitch (Gregersen et al., 1999, 2001; Theusch et al., 2009; Theusch and Gitschier, 2011), congenital amusia (Peretz et al., 2007), and music perception (Drayna et al., 2001; Pulli et al., 2008; Ukkola et al., 2009; Ukkola-Vuoti et al., 2013; Oikkonen et al., 2015). In a study assessing non-musicians, individuals with superior musical ability showed enhanced neural encoding of speech. Moreover, they were less susceptible to noise in a similar way to what appeared in professional musicians (Mankel and Bidelman, 2018). Swaminathan and Schellenberg (2018) examined relationships among musical training and non-musical factors and musical ability to find a marker for musical competence. In this study, non-musical factors such as socioeconomic status, short-term memory, general cognitive ability, and personality were indirectly associated with the musical ability along with the musical training, suggesting that the musical competence would be established by complex interactions between nature and nurture traits. It seems difficult to make a conclusion of nature vs. nurture debate at this point. To clarify the issue, further studies are necessary to compare multiple factors relating to the musical ability in a large group of musicians.

#### N1/P2 Correlation With Behavioral Performance and Musical Training Experience

In our study, N1 is correlated with the perceptual change to frequency information in musician whereas the duration of musical training is related to P2. The lack of a consistent N1 relationship with musical training may be accounted for by the notion that N1 is related to neural processing for frequency information in sound rather than a musical experience. In particular, the relationship between N1 and behavioral performance was found between frequency discrimination thresholds in noise and N1 amplitudes to frequency change (see Figure 6). This finding is related to the previous finding that spectral processing is associated to sound perception in noise (Fu et al., 1998; Won et al., 2007), and difficulty with sound in noise has been attributed to a reduction in the ability to distinguish acoustic signals from noise (Gaudrain et al., 2007). For P2, we found that the amplitude increased with longer duration of musical training but not with age at the onset of the training (data not shown). Relationships between musical training and P2 evoked by auditory stimuli have been reported in previous studies on adults (Atienza et al., 2002; Shahin et al., 2003; Choi et al., 2014) as well as children (Shahin et al., 2003, 2004). Moreover, the P2 amplitude elicited by musical tones is correlated with musical training (Choi et al., 2014). These results suggest that continuous musical training may help to maintain cortical synaptic plasticity regardless of when musical training started. Meanwhile, a previous study comparing behavioral thresholds for speech discrimination and objective/cognitive properties has shown that the P2 threshold is associated with cognitive factors such as non-verbal IQ but not with musical experience (Boebinger et al., 2015); the authors suggested that the musician’s advantage may be accounted for by co-variation in higher-order cognitive factors with musicianship. To better understand the complex relationships among musical training and cognitive and perceptual processing, more studies are necessary to compare perceptual measures of sound processing, cortical activity, and cognitive factors interconnected through both bottom-up and top-down auditory pathways.

### Asymmetrical Hemispheric Activation to Frequency Change

We investigated whether hemispheric asymmetry in the processing of frequency change exists at the cortical level. The findings from the N1/P2 dipole source analysis showed that source activation in response to frequency changes was greater in the right hemisphere than in the left hemisphere. This result is consistent with previous reports (Shahin et al., 2003, 2007; Dimitrijevic et al., 2008; Pratt et al., 2009; Okamoto and Kakigi, 2015) showing that the processing of frequency information is lateralized to the right hemisphere. The right hemisphere dominance for the processing of frequency change seems to be based on fundamental brain mechanisms that are closely related to the functional specialization of the right hemisphere for pitch perception (Zatorre and Belin, 2001). Research on a large sample of musicians has reported that the musicians were sensitive to pitch change and their behavioral sensitivity was associated with the right-ward asymmetry for pitch processing (Schneider et al., 2005). Moreover, a lesion-related study reported abnormal pitch discrimination in patients who had undergone the removal of the right Heschl’s gyrus (Johnsrude et al., 2000). Zatorre and Belin (2001) also confirmed that spectral processing recruits anterior superior temporal regions bilaterally, with greater activation in the right hemisphere (Zatorre and Belin, 2001).

By comparing the left- and right-hemispheric activities separately in musicians and non-musicians, we found that the dipole source activity in musicians evoked by frequency changes was larger than that in non-musicians in both hemispheres. Increased bilateral engagement of the hemispheres in the musician was mainly attributed to the group difference, and the effects of musical training on hemispheric reorganization were only observed for the P2 dipole. Indeed, increased bilateral hemispheric activation following long-term musical experience has previously been reported. Using near-infrared spectroscopy, Gibson et al. (2009) found greater bilateral frontal activity in musicians compared to non-musicians during a cognitively demanding task; they suggested that extensive musical experience yields the symmetrical activities in the musicians. Also, Tremblay et al. (2009) reported that short-term auditory training evoked a different pattern of hemispheric asymmetry such that the P2 dipole sources to training-specific stimuli increased in the left hemisphere. This is consistent with our results showing that musical training enhances cortical activity in the left hemisphere. Furthermore, all of the musicians except for the vocalists in our study require both hands to play their instruments. Musicians can incorporate auditory feedback to play instruments and appropriately alter their motor response in both hands in a very short period. Given that this auditory-motor interaction interplays between the left and right hemispheres, this process may strengthen the direct connections between the hemispheres (reviewed inZatorre et al., 2007).

## Conclusions

In the present study, we showed that the effect of frequency change was more apparent for N1, while P2 responses are closely related to musical training. An enhanced N1 response to frequency changes is associated with better frequency discrimination whereas P2 responses are positively related to the duration of musician training, indicating training-induced cortical plasticity. Also, musicians had more robust P2 source activation in both hemispheres, which indicates musical experience may alter the hemispheric lateralization for processing of frequency change more symmetrically. Given that enhanced P2 activity with frequency change reflects changes in the summation of postsynaptic field potentials in the auditory cortex, our findings infer that neural plasticity evoked by long-term musical training can alter the cortical representation of a change in frequency even when passively listening to sounds. In future studies, we will examine the cortical activity to frequency change with noise-masking to compare with quiet listening to define a neural overlap between pitch perception and sound in noise perception. Also, the effect of attention on spectral processing is worth investigating in that the selective attention in musicians increases the neural encoding of sound and suppresses background noise to enhance their speech-in-noise perception ability (Strait and Kraus, 2011).

## Data Availability Statement

The raw data supporting the conclusions of this article will be made available by the authors, without undue reservation.

## Ethics Statement

The study involving human participants were reviewed and approved by the ethics committee of the Hallym University Sacred Hospital. The patients/participants provided their written informed consent to participate in this study.

## Author Contributions

JL and J-HH contributed to the conception, design of the study, and wrote the manuscript. JL performed the experiment and statistical analysis. JL, J-HH, and H-JL contributed to manuscript revision, read, and approved the submitted version.

## Conflict of Interest

The authors declare that the research was conducted in the absence of any commercial or financial relationships that could be construed as a potential conflict of interest.
